# Predictive Value of Blood Urea Nitrogen/Albumin Ratio in Mortality in Moderate to Severe COVID-19 Patients: A Retrospective Observational Analysis

**DOI:** 10.7759/cureus.48416

**Published:** 2023-11-06

**Authors:** Nidhi Kaeley, Shiana Singh, Prakash Mahala, Suman Choudhary, Utkarsh P. Singh

**Affiliations:** 1 Emergency Medicine, All India Institute of Medical Sciences, Rishikesh, Rishikesh, IND; 2 Microbiology, All India Institute of Medical Sciences, Rishikesh, Rishikesh, IND

**Keywords:** hospital mortality, emergency medicine, patients with covid-19, mortality marker, bun/albumin ratio

## Abstract

Background: The coronavirus disease of 2019 (COVID-19) pandemic was associated with a high mortality rate. It posed a formidable challenge to healthcare systems worldwide. In this study, we evaluated the predictive value of the blood urea nitrogen (BUN)/albumin ratio as a mortality marker in patients with moderate to severe COVID-19 infection in the emergency department (ED).

Methodology: A retrospective evaluation of 352 patients with moderate to severe COVID-19 infections was conducted. Out of the 352 patients, 183 (51.99%) were discharged and 169 (48.01%) succumbed. Comprehensive demographic, clinical, biochemical, and haematological data was compiled for each patient. BUN to albumin ratios were determined for all patients, and all data were compared between survivors and non-survivors.

Results: This study included 352 patients. The average length of stay in the hospital was 13 days. In the survivor group, the median BAR value was 0.012, but in the non-survivor group, it was 0.022 (p > 0.001). Also, it was determined that the differences in creatinine, BUN, and albumin between the two groups were statistically significant. The median BAR value was significantly higher in the non-survivor group (0.022 [0.014-0.033]) as opposed to the survivor group. Also, the median values of creatinine were higher and albumin was lower in the non-survivor group. This difference was statistically significant.

Conclusion: The BUN/albumin ratio can be utilized as a marker of mortality in patients with COVID-19 infection presenting to the emergency department.

## Introduction

Coronavirus disease (COVID-19), an infectious viral illness, was first reported in December 2019 in the Chinese district of Wuhan. The World Health Organization (WHO) labelled it a pandemic in 2020 [1]. According to the WHO, the total number of documented cases of COVID-19 until January 2023 was 659,108,952, with 6,684,752 fatalities globally. The reported COVID-19 mortality rate is around 2%. However, some studies have found a mortality rate as high as 48.4 percent among hospitalized patients with a severe COVID-19 infection [2]. COVID-19 poses a humongous challenge to emergency departments (EDs) around the world. The largest obstacle was the growing shortage of beds and doctors. This obstacle can be addressed with the use of biomarkers that are freely accessible and can easily and rapidly predict the mortality and severity of COVID-19 patients brought to the emergency department.

Overcrowding in the ED is a global issue [3-4]. There have been several studies on the negative impacts of overcrowding and how to alleviate them [5-6]. We may overcome this obstacle by predicting the patient's prognosis early and initiating the necessary treatment promptly. It has been shown that specific serum biomarkers can predict mortality and reflect the severity of pneumonia [7]. Numerous laboratory investigations, such as D-dimer, c-reactive protein (CRP), lactate dehydrogenase (LDH), ferritin, procalcitonin (PCT), and cytokine assays, especially interleukin-6 (IL-6), have been investigated for their prognostic and therapeutic role in COVID-19 patients. The aforementioned investigations, although demonstrated to have good predictive ability, do have their own limitations. First, these biomarkers are not specific to COVID-19 and may be raised in a multitude of conditions, such as bacterial infections, autoimmune diseases, and malignancy. Second, their levels may be influenced by other factors such as age, gender, obesity, and smoking. Finally, the levels of these biomarkers do not reflect the extent of underlying organ dysfunction. In lieu of the drawbacks posed by the biomarkers stated, studies were conducted to evaluate other biomarkers in COVID-19 infection, especially under the circumstances of limited medical resources. Among these, the BUN/albumin ratio has been of significant interest.

Albumin is the most abundant plasma protein in humans [8]. The distribution of albumin between intravascular and extravascular compartments is altered by critical illness. There are also variations in the rates of protein production and breakdown of albumin during critical illness [9]. Early in the course of a critical illness, the serum albumin levels decrease due to increased capillary leakage, often significantly and not rising until the time of recuperation from the sickness [10]. It has been demonstrated that its concentration is decreased in individuals with pneumonia [11], acute coronary syndrome [12], pancreatitis [13], and sick geriatric patients [14].

Blood urea nitrogen, often known as BUN, is a nitrogenous waste product that is produced during the process of protein metabolism. Blood urea nitrogen (BUN) is an essential measure of the relationship between a patient's renal health and nutritional status. It has also been linked to mortality in various diseases [15]. In addition to its role in determining renal function, BUN is a surrogate test for predicting persisting organ failure 48 hours after hospital admission [16-17]. The BUN can independently predict death in critically ill patients admitted to the intensive care unit, according to multicentre research (ICU) [18]. Furthermore, the bun to serum albumin ratio (BAR) was presented as a significant predictor of mortality in a variety of conditions, such as gastrointestinal haemorrhage, community-acquired pneumonia, etc. [7,19-20].

Consequently, biomarkers such as the ratio of BUN to albumin (BAR) can be utilized as an inflammatory measure for predicting the mortality of patients with moderate to severe COVID-19 infection in the emergency department. In the present study, we aim to determine the prognostic significance of the BAR.

## Materials and methods

Study design

A retrospective, single-centre observational research study was conducted over a period of three months, from March 10 to June 11, 2021. The Institutional Ethics Committee, All India Institute of Medical Sciences, Rishikesh (AIIMS/IEC/21/109; 04/03/2021), approved this study.

Study population

Patients over the age of 18 with at least one positive reverse transcriptase polymerase chain reaction (RT-PCR) test for COVID-19 with a moderate to severe type of pneumonia (SpO_2_ <94%) who were admitted to the ED were included. However, patients with a prior history of chronic renal failure (CRF) with maintenance haemodialysis, chronic liver disease, secondary vasculitis, and those who sought leave against medical advice (LAMA) were excluded from the study.

Study procedure

Comprehensive demographic, clinical, biochemical (liver function test and kidney function test), and haematological (complete hemogram) data were compiled for each individual patient with a moderate to severe COVID-19 infection, along with outcome parameters such as mortality, need for invasive ventilation, and discharge. Data was taken from the respective patient files and online hospital records. The ratio of BUN to albumin (BAR) was calculated for each patient by dividing BUN (mg/dL) by albumin (g/dL). In-hospital mortality was the primary endpoint. Patients were assigned to one of two groups: the survivor group or the non-survivor group, depending on whether they were effectively treated for COVID and discharged from the hospital or did not survive the disease.

Statistical analysis

Using the SPSS software (IBM Corp., released 2011). In IBM SPSS Statistics for Windows, version 29.0 (Armonk, NY: IBM Corp.), the data were analysed. Categorical data were expressed as frequency and percentage. Quantitative data were expressed as the mean and standard deviation (SD). Those quantitative variables that did not follow a normal distribution were expressed as the median and range. An independent t-test and a Mann-Whitney U test (rank-sum test) were used to compare quantitative variables between the survival and non-survival categories of the patient. A chi-square or Fisher's exact test was used to check the association between survival and categorical variables. Receiver operating characteristic (ROC) curve analysis was carried out to find the cut-off value for non-survival in COVID-19 patients for BAR. Using this cut-off, the sensitivity, specificity, positive predictive value (PPV), negative predictive value (NPV), and odds ratio (OR) were obtained. A p-value less than 0.05 is deemed statistically significant.

## Results

Descriptive statistics

Taking into account the inclusion and exclusion criteria, a total of 352 patients were included in this study, with 74.15% males and the rest females. The median hospitalization duration was 13 days. The majority of patients reported having a fever (94.31%), followed by shortness of breath (76.42%), and coughing (75.28%). A minor percentage of patients (8.52%) also reported myalgia, diarrhoea, and/or anosmia. At least one comorbidity was present in the medical history of 44.32% of patients, with hypertension (40.34%) being the most prevalent, followed by diabetes (15.05%), coronary artery disease (CAD) (9.37%), and chronic obstructive pulmonary disease (COPD) (1.13%). In-hospital mortality occurred in 48.01% of patients (non-survivor group) and did not occur in 51.99% of patients (survivor group). Table 1 depicts the descriptive data of the study.

**Table 1:** Demographic and clinical parameters of the patients with moderate to severe COVID-19 pneumonia SBP: systolic blood pressure, DBP: diastolic blood pressure, MAP: mean arterial pressure, T2DM: type 2 diabetes mellitus, COPD: chronic obstructive pulmonary disease, CRF: chronic renal disease, GCS: Glasgow Coma scale, HFNC: high-flow nasal cannula.

Parameters	Survivors (n=183)	Non-survivors (n=169)	p-value
Age (years)	51.99 ± 15.71	72.01 ± 13.67	<0.001
Male (%)	139 (75.9%)	122 (72.1%)	0.422
Female (%)	44 (24.04%)	47 (27.8%)	
SBP (mm of Hg)	124.60 ± 17.61	17.51 ± 20.88	0.383
DBP (mm of Hg)	78.50 ± 49.56	73.81 ± 14.11	0.265
MAP (mm of Hg)	95.1 ± 33.99	91.94 ± 14.23	0.411
Heart rate (beats per minute)	93.38 ± 15.44	95.72 ± 14.46	0.139
Breaths (per minute)	22.78 ± 6.51	28.67 ± 8.49	0.002
Saturation (%)	91.1±6.32	80.81 ± 6.23	<0.001
Hypertension (%)	44 (24.04%)	98 (57.98%)	0.002
T2DM (%)	30 (16.39)	23 (13.6)	0.9
Coronary artery disease (%)	16 (8.7)	17 (10.05)	1
COPD (%)	1(0.5)	3 (1.77)	1
CRF (%)	55 (30.05)	65 (38.36)	0.8
Length of stay (days)	13.81 ± 7.82	3.88 ± 335.7	<0.001
Non-invasive ventilation (%)	15 (8.19)	45 (26.62)	<0.001
Invasive ventilation (%)	0	142 (84.03)	<0.001
HFNC (%)	14 (7.6)	123 (72.78)	<0.001
Need for vasopressor (%)	4 (2.18)	61 (36.09)	<0.001

Other variables and patient outcomes

Various other factors were studied for their prognostic role, as shown in Table 1. It was found that age, oxygen saturation at admission, tachypnea, need for non-invasive ventilation (NIV), high-flow nasal cannula (HFNC), invasive ventilation, need for vasopressor support, and longer hospital stays were significantly associated with mortality. It was observed that comorbidities such as hypertension, diabetes, CRF, CAD, and COPD were also associated with greater mortality.

BAR and patient outcomes

Serum investigations and BAR values in the survivor and non-survivor groups are shown in Table 2. The median BAR value in the non-survivor group (0.022) was higher as compared to the survivor group (0.012). This was assessed to be statistically significant (p<0.001). The difference in creatinine, BUN, and albumin between the two groups was also found to be statistically significant.

**Table 2:** Mean values of biomarkers of the patients with moderate to severe COVID-19 pneumonia HB: haemoglobin, CREAT: creatinine, BUN: blood urea nitrogen, B/A ratio: BUN/albumin ratio.

Parameters	Outcome		p-value
	Survivors	Non-survivors	
HB (g/dl)	12.34 (11.07–14.19)	12 (10–14.2)	0.899
CREAT (µmol/l)	1.05 (0.93–1.90)	1.26 (1.02–2.10)	0.001
BUN (mg/dl)	47.30 (32.48–65.20)	65.20 (46.30–88.0)	<0.001
ALBUMIN (g/dl)	4.10 (3.18–4.95)	3.10 (2.4–4.2)	<0.001
B/A ratio	0.012 (0.008–0.017)	0.022 (0.014–0.033)	<0.001

ROC analyses

**Figure 1:**
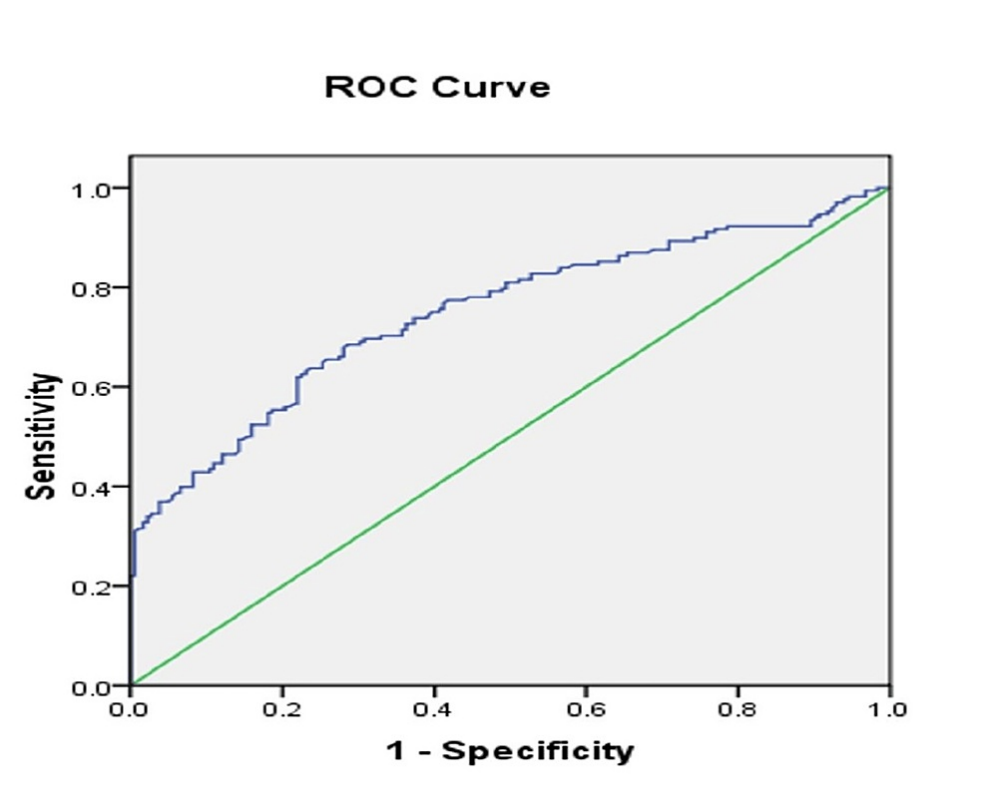
ROC analysis performed to establish the predictive power of BAR for in-hospital COVID-19 mortality

The ROC analysis performed to establish the predictive power of BAR for in-hospital COVID-19 mortality is shown in Figure 1. At a cut-off value of 0.017, BAR predicted mortality with a sensitivity, specificity, and accuracy of 63.7%, 76.4%, and 70.3%, respectively.

## Discussion

In this study, we investigated the relationship between various clinical parameters and mortality in COVID-19 patients. The findings shed light on the critical role that age and several other clinical factors play in determining patient outcomes.

Our study revealed a significant correlation between age and mortality among COVID-19 patients. The mean age of survivors was 51.99 ± 15.71 years, significantly lower than that of non-survivors at 72.01 ± 13.67 years (p < 0.001). In the geriatric population, immune senescence is commonly cited as the underlying cause of increased susceptibility to infections in the elderly. Immunosenescence is a term used to describe the age-related decline in the immune system's function and effectiveness. As individuals age, their immune response undergoes various changes, making the elderly population more vulnerable to infections and diseases [21-22]. This finding is consistent with previous research that identified advanced age as a crucial risk factor for severe COVID-19 outcomes [23-24].

The study also explored respiratory parameters, including respiration rate and saturation levels, as potential predictors of mortality. Notably, non-survivors exhibited higher respiration rates (28.67 ± 8.49 per minute) compared to survivors (22.78 ± 6.51 per minute), showing a statistically significant difference (p = 0.002). Additionally, saturation levels were significantly lower in non-survivors (80.81 ± 6.23%) than in survivors (91.1 ± 6.32%) (p < 0.001). These results indicate that respiratory distress is closely associated with unfavourable COVID-19 outcomes [25-26].

Our study investigated the presence of hypertension and type 2 diabetes mellitus (T2DM) as potential comorbidities contributing to mortality. We observed that non-survivors had a higher prevalence of hypertension (57.98%) compared to survivors (24.04%) (p = 0.002). However, no statistically significant difference was found in the occurrence of T2DM between the two groups (p = 0.9). Elevated uncontrolled systolic blood pressure (SBP) could potentially exacerbate the severity of the disease, as it is linked to hypertension-mediated subclinical organ damage (HMOD), including vascular remodelling. This process may further deteriorate endothelial function and lead to endothelial damage and endotheliitis caused by the SARS-CoV-2 infection. These results align with previous studies highlighting the role of hypertension in exacerbating COVID-19 outcomes [27-29].

The study examined the length of stay and the need for various respiratory support interventions. Non-survivors had significantly shorter hospital stays (3.88 ± 3.57 days) compared to survivors (13.81 ± 7.82 days) (p < 0.001). Moreover, a substantial number of non-survivors required non-invasive ventilation, invasive ventilation, HFNC, and vasopressors compared to the survivors (all p < 0.001). These findings emphasize the critical role of timely and appropriate respiratory support in influencing COVID-19 patient outcomes [30-31].

The objective of this study was to assess the role of BAR as a prognostic marker for in-hospital mortality in COVID-19 patients. The study divulged that albumin, BUN, and BAR were significantly correlated with in-hospital mortality, as non-survivor group patients had lower albumin, higher BUN, and consequently, high BAR values compared to the survivor group. This finding is similar to previous studies where it was found that the BUN to albumin ratio was significantly higher in severe COVID-19 cases compared to mild cases and was associated with an increased risk of mortality [14,32-33]. There have been several studies comparing BUN with other biomarkers used in COVID-19. In a retrospective study of 514 COVID-19 patients, researchers found that BAR was a better predictor of mortality than CRP, procalcitonin, or LDH levels [34]. Another study of 198 COVID-19 patients found that the BAR was a better predictor of disease severity than NLR or CRP levels [35]. Finally, a study of 161 COVID-19 patients found that the BAR was a better predictor of mortality than D-dimer or ferritin levels [36].

Limitations and future directions

The present study had certain limitations. First, the retrospective nature of the research design may introduce inherent biases. Second, the sample size might not fully represent the entire COVID-19 patient population. Third, although our study adds to the scarcity of literature regarding BAR, we could not compare BAR to other markers shown to be prognostically beneficial in COVID-19. It is important to note that the BAR should not be used in isolation as a prognostic marker, as other factors such as age, comorbidities, and disease severity also play important roles in COVID-19 prognosis. Additionally, the BAR may be affected by various factors such as hydration status, liver function, and medications, which can complicate its interpretation. Despite the valuable insights gained from this study, there are certain limitations to consider. Future studies with larger, more diverse cohorts are warranted to validate these findings further.

## Conclusions

In conclusion, our study underscores the significance of age and various clinical parameters in determining COVID-19 patient outcomes. There are a multitude of advantages to using BAR as a prognostic factor in COVID-19. The BAR is a non-invasive marker that can be calculated from a simple blood test, which is less invasive than other markers such as computed tomography scans or bronchoscopy. This makes it a safer and less burdensome marker for patients. The BUN and albumin tests are widely available and routinely used in clinical practice, making the BUN to albumin ratio a marker that can be easily calculated in many healthcare settings.

It is a simple and cost-effective marker, which makes it convenient for clinicians to use in the management of COVID-19 patients. The BUN to albumin ratio reflects both kidney function (BUN) and nutritional status (albumin), both of which are important factors in the COVID-19 prognosis. By using a single ratio that reflects multiple aspects of patient health, clinicians may be able to obtain a more comprehensive picture of a patient's overall health status. By identifying COVID-19 patients at increased risk of disease severity and mortality, the BUN to albumin ratio may help clinicians make more informed clinical decisions, such as earlier initiation of intensive care or more aggressive treatment strategies. In summary, while the BAR has shown promise as a potential prognostic marker in COVID-19, further research is needed to determine its clinical utility and identify its optimal cut-off values for predicting disease severity and mortality risk in COVID-19 patients.
